# Supplementary material for: FERN – a Java framework for stochastic simulation and evaluation of reaction networks
Source: BMC Bioinformatics. 2008 Aug 29;9:356. doi: 10.1186/1471-2105-9-356 (PMC2553347; doi:10.1186/1471-2105-9-356)
Supplement: Additional file 1 — FERN distribution, Version 1.3. This archive contains the FERN source code and binaries as well as documentation and example models in FernML and SBML. [file 1471-2105-9-356-S1.zip › fern/doc/javadoc/index-files/index-4.html]

D-Index


---


|  |  |  |
| --- | --- | --- |
| **Overview** \| Package \| Class \| Use \| **Tree** \| **Deprecated** \| **Index** \| **Help** | |  |
| **PREV LETTER**   **NEXT LETTER** | **FRAMES**    **NO FRAMES**     **All Classes** |  |


A B C D E F G H I J L M N O P R S T U V 

---


## **D**

**DecayingDimerizingHistogramDistances** - Class in fern.example: Demonstration of performance and accuracy differences of the different simulation algorithms. **DecayingDimerizingHistogramDistances()** - Constructor for class fern.example.DecayingDimerizingHistogramDistances: **DecayingDimerizingInteractive** - Class in fern.example: Demonstration of performance and accuracy differences of the different simulation algorithms. **DecayingDimerizingInteractive()** - Constructor for class fern.example.DecayingDimerizingInteractive: **DecayingDimerizingPlots** - Class in fern.example: **Deprecated.** **DecayingDimerizingPlots()** - Constructor for class fern.example.DecayingDimerizingPlots: **Deprecated.** **DefaultController** - Class in fern.simulation.controller: The probably most common implementation of an `SimulationController`. **DefaultController(double)** - Constructor for class fern.simulation.controller.DefaultController: Creates the controller for a given time where the simulation has to stop. **defaultStyle** - Variable in class fern.tools.gnuplot.GnuPlot: **DependencyGraph** - Class in fern.simulation.algorithm: Implements a dependency graph. **DependencyGraph(Network)** - Constructor for class fern.simulation.algorithm.DependencyGraph: Creates the dependency graph for a given network **describe()** - Method in class fern.cytoscape.CytoscapeVisualizer: **detect()** - Method in class fern.analysis.AutocatalyticNetworkDetection: Performs the detection algorithm. **dfs(int[], int[], NetworkSearchAction)** - Method in class fern.analysis.AnalysisBase: Performs a depth first search starting at the given sources (which means the contents of `speciesSource` and `reactionSource` are the initial content of the stack. **directMCTau(double)** - Method in class fern.simulation.algorithm.GillespieEnhanced: obtains a random (but following a specific distribution) timestep as described by the direct method in chapter 5A page 417ff **directMCTau(double)** - Method in class fern.simulation.algorithm.GillespieSimple: obtains a random (but following a specific distribution) timestep as described by the direct method in chapter 5A page 417ff **directMCTau(double)** - Method in class fern.simulation.algorithm.HybridMaximalTimeStep: obtains a random (but following a specific distribution) timestep as described by the direct method in chapter 5A page 417ff **Dsmts** - Class in fern.example: Perform a series of tests (refer to http://www.calibayes.ncl.ac.uk/Resources/dsmts). **Dsmts()** - Constructor for class fern.example.Dsmts: **dumpMathTree(MathTree)** - Static method in class fern.tools.NetworkTools: Dumps the MathTree to stdout. **dumpMathTree(MathTree, Writer)** - Static method in class fern.tools.NetworkTools: Dumps the math tree to the given `Writer`. **dumpNetwork(Network)** - Static method in class fern.tools.NetworkTools: Dumps the network to stdout. **dumpNetwork(Network, Writer)** - Static method in class fern.tools.NetworkTools: Dumps the network to the given `Writer`.

---


|  |  |  |
| --- | --- | --- |
| **Overview** \| Package \| Class \| Use \| **Tree** \| **Deprecated** \| **Index** \| **Help** | |  |
| **PREV LETTER**   **NEXT LETTER** | **FRAMES**    **NO FRAMES**     **All Classes** |  |


A B C D E F G H I J L M N O P R S T U V 

---
